# NHS healthcare worker experiences of moral injury: a follow-up qualitative study in England using reflexive thematic analysis

**DOI:** 10.1136/bmjopen-2025-115402

**Published:** 2026-08-18

**Authors:** Erin Higginbottom, Friederike Stoeck, Sharon Stevelink, Siobhan Hegarty, Rosalind Raine, Anne Marie Rafferty, Neil Greenberg, Simon Wessely, Hassan Rahman, Danielle Lamb

**Affiliations:** 1 University College London, London, UK; 2 King’s College London, London, UK

**Keywords:** MENTAL HEALTH, QUALITATIVE RESEARCH, Occupational Stress

## Abstract

**Abstract:**

**Background:**

Moral injuries (MIs) may result from individuals being exposed to potentially morally injurious events (PMIEs). PMIEs have been a feature of work within much of the National Health Service (NHS) for many years, and the understanding of MI is now more commonplace, having been highlighted during the COVID-19 pandemic; however, understanding of how such events affect workers over the long term is lacking.

**Objective:**

To understand the development of healthcare workers’ (HCWs) experiences of PMIEs over a period of 3 years.

**Design and participants:**

We carried out follow-up semistructured qualitative interviews with 15 NHS staff who were previously interviewed on the same topic in 2021. All interviews were recorded, transcribed and analysed using reflexive thematic analysis following an inductive approach.

**Results:**

Three main themes and six subthemes were identified: (1) internal context and subjective experiences, (1.i) control in a rigid system, (1.ii) psychosocial dynamics, (2) external context and the moral cost of culture, (2.i) organisational culture, (2.ii) sources of MI, (3) responses to PMIEs, (3.i) maladaptive responses, (3.ii) adaptive responses. Key issues identified included perceptions of insufficient control over their work and work environment, low psychological safety and perceived misunderstanding of the value of their work from patients and colleagues, leading to a challenging organisational culture. We found that in the 3 years between the interviews many staff reported developing ‘secondary’ feelings of betrayal as a result of perceived ongoing governmental neglect of recognition for HCWs’ sacrifices during the pandemic and a lack of acknowledgement of governmental mistakes made in the acute period.

**Conclusion:**

Findings show an interplay between individual and organisational factors that HCWs faced while working in the NHS, underlining the need for wide-ranging intervention strategies. Since the HCWs’ original interviews in 2021, a secondary betrayal has developed, emphasising the need for interventions that can restore trust in the system.

**STRENGTHS AND LIMITATIONS OF THIS STUDY**

- A key limitation is that we achieved follow-up participation of only 50% and consequently, our sample reflects only those still working, which may underestimate the true extent of moral injury (MI) in the National Health Service, and the adaptive responses identified may be over-represented.
- Another limitation is that, while some participants reported experiencing potentially morally injurious events, they struggled to provide examples and while probing mitigated this, future research could encourage reflection before interviews to support richer responses, for example, by using storytelling, diaries.
- A further limitation is that we used an existing accepted definition of MI to enable comparison to previous research, which may have biased the answers given by respondents.
- A strength is that our semistructured, participant-led approach respected the subjective nature of MI and did give space for participants to self-define their experiences.
- Another strength is that the follow-up design offered insight into the trajectories of MI and established the identification of a secondary betrayal.

## Introduction

 Moral injury (MI) refers to the lasting guilt, shame, sense of betrayal or loss of trust that can occur when institutionally or socially required behaviour conflicts with personal beliefs.^1^ In healthcare, potentially morally injurious events (PMIEs) arise when (1) someone commits or fails to prevent an act that goes against their moral values, (2) witnesses another’s moral transgressions or (3) feels betrayed, often by those in positions of power.^2^ Examples during the COVID-19 pandemic included rationing healthcare resources, preventing family members being present for patient deaths, staff being expected to provide care without adequate personal protective equipment (PPE) and being unable to provide usual standards of care. Since the pandemic, examples of morally injurious events include being pressured to ‘catch up’ on delayed care by providing faster, lower quality care and being forced to turn away patients who do not meet service criteria due to the volume of referrals (eg, in mental health services). PMIEs can cause moral distress, which we define as the psychological discomfort that can arise from inability to act in accordance with one’s values, and may or may not develop into MI, which, as above, refers to a deeper, more enduring harm to one’s moral identity.

MI is associated with strong emotions such as guilt, anger or shame, which may lead to mental disorders such as post-traumatic stress disorder (PTSD), depression or self-harm or suicidal behaviours,^3^ but is not itself a clinically diagnosable condition. In healthcare workers (HCWs), a global systematic review of 18 studies found that burnout, trauma, compassion fatigue, depression, anxiety and PTSD were all positively associated with MI.^4^ An umbrella review found the prevalence of self-reported depression and anxiety disorders among HCWs in the early stages of the pandemic was 25%.^5^ MI likely contributed to this, reflecting the psychological toll of COVID-19-related PMIEs.

### Experiences of MI during the acute phase of COVID-19

In the year to December 2020, COVID-19 accounted for 12.3% of all deaths (n=69 771) in England and Wales.^6^ By January 2021, high hospital and intensive care unit (ICU) admissions intensified pressure on HCWs, who faced PPE and resource constraints.^7^

Several studies demonstrated HCWs’ exposure to PMIES during COVID-19. For example, a reflexive thematic analysis (RTA) of 16 National Health Service (NHS) workers found frequent betrayal-based MI, with staff feeling abandoned by leadership over PPE issues.^8^ Similarly, a USA-based phenomenological study found nurses felt betrayed by employers who misled the public about PPE availability.^9^ Our prior research in 2021^7^ found strained relationships between HCWs and management: frontline workers felt managers were absent, while managers felt guilty and similarly unsupported by their own managers.

### The current climate of the NHS

In May 2023 the WHO declared COVID-19 was no longer a public health emergency.^10^ Studies have investigated the relationship between PMIEs related to the onset of the pandemic and the development of MI in HCWs, but there is a gap in understanding the ‘normalisation’ period, characterised by the shift from pandemic-related emergency measures as HCWs returned to more routine work. This period has also been referred to as the ‘living with Covid’ phase.^11^ Cross-sectional research from China during the normalisation period indicated that prevalence of self-reported symptoms of depression and anxiety among HCWs were 21.3% and 12.7%, respectively.^12^ This finding indicated that the reduction in probable depression was relatively small compared with the drop in anxiety symptoms, as an umbrella review conducted during the acute period had found prevalence of 25% for both conditions.^5^ This may suggest that, over time, HCWs became more confident (ie, less anxious) about working with infection-control protocols. It is unclear whether the relatively small drop in the rates of probable depression reflects HCWs remaining hopeless about the long-term consequences of the pandemic, or a return to the higher rate of probable depression which existed before the pandemic. While these results offer indirect insights into changes in mental health across the pandemic, robust longitudinal data is needed to measure a trajectory.

In the UK, as the pandemic progressed, vaccination roll-outs increased, ICU admissions decreased and COVID-19 deaths declined.^13^ However, HCWs have since faced a patient backlog and increased demand from patients, characterised by heightened awareness of and anxiety about health. In May 2025 there were 7.4 million cases on the NHS waitlist for care.^14^ In this new climate, the nature and impact of PMIEs explored during the early stages of the pandemic^7–9^ may have changed, especially given developments such as the widespread HCW pay disputes,^15^ with many reporting feeling undervalued and let down by government. Given the limited available research into MI among HCWs in the postpandemic period, further investigation is needed to understand the development of MI experienced by HCWs. Better understanding of MI in HCWs will identify opportunities for prevention (where possible) and support to be provided where necessary.

### Aims and objectives

This study aimed to follow-up an established cohort of HCWs who were interviewed in 2021 about their experiences of PMIEs during COVID-19 and the impact of these experiences on their well-being. This study aimed to identify what, if anything, had changed in relation to their experiences of PMIEs and their mental health since then.

## Methods

### Design

Follow-up interviews were conducted with 15 HCWs previously interviewed in November/December 2021.^7^ All interviews are part of the NHS CHECK study, a prospective cohort study that investigates the well-being of NHS staff during and beyond the COVID-19 pandemic.^16 17^ The rationale for conducting individual interviews rather than focus groups was to enable participants to speak more freely about their experiences than they might have felt able to in a group discussion. The interviews were analysed using RTA,^18^ adopting an inductive approach.

### Patient and public involvement and engagement

The NHS CHECK patient and public involvement and engagement advisory group provided input on the design of this study and the interview schedule used to collect data. 

### Participants

Participants (hereafter referred to as ‘HCWs’) were NHS staff who had taken part in interviews in November/December 2021, all of whom had consented to be contacted again for future research. There was a 50% follow-up rate with 15 out of the original 30 HCWs agreeing to participate. The original inclusion criteria were that HCWs had to have: (1) been a frontline clinical staff member working in England during the pandemic; (2) completed the NHS CHECK 6 months follow-up survey; (3) reported that they had experienced a PMIE through the 9-item self-report MI events scale^19^ in the NHS CHECK 6 months follow-up survey, as we were interested specifically in the experiences of those reporting experiences of PMIEs. HCWs were originally chosen from a representative sample of the NHS CHECK study (NHS Trust, age, sex, job role, ethnicity); as with any follow-up study, this research will be biased towards the characteristics of the remaining HCWs who agreed to take part. The HCWs’ demographics are provided in table 1.

**Table 1:** Demographic information of sample (n=15)

Variable	Category	Current sample N=15 n (%)	2021 sample N=30 n (%)
Sex	Male	7 (47)	10 (33)
	Female	8 (53)	20 (67)
Ethnicity	White	10 (67)	18 (60)
	Asian	2 (13)	6 (20)
	Black	2 (13)	2 (7)
	Mixed	1 (7)	3 (10)
Occupation	Clinical	12 (80)	21 (96)
	Non-clinical	3 (20)	1 (4)

### Procedure and materials

HCWs were contacted via the email addresses they provided previously. The participant information sheet was attached, and after the first emails were sent, two follow-up emails were sent to non-respondents. Once HCWs agreed to participate, we provided the digital consent form and scheduled an interview date/time convenient to them, using Microsoft Teams. An updated topic guide (online supplemental file 1) was developed from the topic guide used in the original 2021 interviews (online supplemental file 2). The guide focused on PMIEs HCWs experienced since their original interview in 2021, the impact this had on their work and mental health, and any helpful/unhelpful support given. The interviews were conducted by EH, FS and DL. The interviews were conducted and recorded in Microsoft Teams video meetings, held between 7 May 2024 and 7 June 2024. Teams auto-transcription was used to produce initial transcripts. While listening to the recordings and reading through the transcripts, the data was anonymised and errors corrected, and this process began familiarisation with the data. The transcripts were then imported into NVivo V.14, which was used to support the analysis process.

### Data analysis

RTA was used as it encourages a deep, interpretive and iterative process of analysis, and the researchers engaged in the data through reflecting, reading and re-reading the interview transcripts. The RTA took a critical realist position, acknowledging that the HCWs exist in a reality that they have discussed with us and we are aware of the influences of certain social structures in shaping our understanding of that reality. It is important to consider our knowledge of the themes that were previously constructed in the 2021 study (online supplemental file 3), as this may have influenced our interpretations of the data. For example, there is a possibility of confirmation bias, where more attention is given to the data that confirms the themes in the initial analysis. To mitigate this, a reflexive journal was kept questioning assumptions and judgments, as well as the research team discussing the coding process to share perspectives. The researchers who conducted most interviews and carried out the initial coding process (EH and FS) were not involved in the study in 2021, and this helped to ensure that the coding took an inductive, data-driven approach.

### Reflexivity

The authors include a mixture of women and men, of varying ages (20s–60s), with varying clinical and academic experience, originating from a variety of countries and are all white, and we acknowledge the limitations this may have introduced to our analysis. We attempted to mitigate this by discussing our findings with colleagues from different ethnic groups, and reflecting on whether our interpretations contained unconscious biases. The reflexive journal was helpful in noting our own preconceptions and assumptions about how those working in the NHS at this time might feel about the local and national pressures on themselves and the wider system. For example, several of us have family or friends who work in the NHS, and who have discussed their own feelings of betrayal and mismatch of values. We acknowledge the extensive media coverage of the pressures in the NHS, and that this may have influenced our interpretations of the data, inclining us to notice negative experiences shared with us over positive experiences. Indeed, we note that some HCWs questioned their career choice, and while career doubt may represent a healthy response at the individual level, we have viewed it as maladaptive in this context due to the fact that staff should not be put in positions where they feel their only option is to leave and the implications for the functioning/sustainability of the NHS. Some authors reflected on their existing cynicism about the structure and sustainability of current NHS operations, and how this may have shaped the analysis. All authors value the NHS and several of the authors either work in clinical roles in the NHS themselves, or have close family members or friends who do.

## Results

### Summary

We identified three overarching themes: (1) Internal context and subjective experiences: HCWs’ own psychological perspectives which were impacted by challenging interactions with both colleagues and patients; (2) external context and the moral cost of culture: the organisational culture HCWs were working in, wider governmental betrayal and how their experiences of MI were shaped; and (3) responses to PMIEs: adaptive and maladaptive responses. The themes are outlined in box 1, followed by detailed results. HCWs have been given pseudonyms rather than ID numbers, preserving anonymity while maintaining humanity.

Box 1 **Themes and subthemes**

1. Internal context and subjective experiences.
  - Control in a rigid system.
  - Psychosocial dynamics and fractured connections.
2. External context and the moral cost of culture.
  - Organisational culture.
  - Sources of MI.
3. Responses to PMIEs.
  - Maladaptive responses.
  - Adaptive responses.

MI, moral injury; PMIEs, potentially morally injurious events.

#### Theme 1: internal context and subjective experiences

This theme refers to the psychological landscape of the HCWs and how their personal experiences and perceptions affected their well-being. This theme encompasses their sense of control and the psychosocial dynamics within the NHS, and how this affected HCWs personally.

##### Control in a rigid system

Several HCWs reported a lack of autonomy and linked this to being more likely to experience PMIEs. For example, recruitment of new staff was the cause of tensions, but this was dependent on role and relative positions of power: while those in senior roles who had more ‘breathing space’ could take time for recruitment at the senior level, other HCWs in more junior roles were often left with no choice but to recruit inexperienced starters who were less able to deliver high-quality care.

We’ve needed a workforce and so we've employed people for the sake of it rather than the really good ones who are going to be able to deliver optimal care. (Helena, non-clinical)

HCWs often perceived themselves to have a lack of job control when collaborating with other departments or NHS services. For example, one participant highlighted their lack of control over clinical processes and patient outcomes as her professional input, grounded in established guidelines, was dismissed by another service she referred to. The disregard for her suggestions signified a rigid system where HCWs felt they had no control in advocating for what they believed was in the patients’ best interest.

He’s not on the treatments advocated by the NICE guidelines, can you see him, and it was like no, we’ve made a decision we’re not going back on it. (Maria, clinical)

Most HCWs acknowledged that formal support delivered by their Trusts was available, however perceived this to be inaccessible. Within systemic constraints, many felt they lacked power to use these sources of support due to workload pressures, with insufficient time to fit in self-care. Alice (clinical) described her Trust advertising the support but stated, “where do you find the time”.

Although not typical, two HCWs reflected an internal locus of control, putting the onus on themselves to engage in support. Jessica expressed the idea that knowledge equalled power, and awareness itself was seen as a form of control.

If people know this stuff is out there to utilise, they’ll - it’s their choice whether they utilize it or not. (Jessica, clinical)

##### Psychosocial dynamics and fractured connections

This subtheme encapsulates how the psychological experiences of the HCWs were shaped by their interactions with colleagues and patients. Many HCWs perceived that they were underappreciated and misunderstood by patients, colleagues or managers, which affected their motivation/well-being. Nearly half of the HCWs discussed their frustration with patients’ perceptions of the NHS and addressed the discrepancy between patient expectations and the reality of medical urgency. One participant, who described being less stressed and more positive, reported using their communication skills to encourage patients “to understand what’s happening inside the NHS” (Bronagh, non-clinical). Discussions with patients to help them understand what it was like to work in the NHS were seen as a way to build empathy. While some had conversations with patients, others demonstrated through industrial action. The act of demonstrating publicly, despite feeling uneasy about it, reflected Myles’ internal struggle between wanting to maintain professional integrity/loyalty and feeling compelled to advocate for better working conditions.

I’m not proud to have gone on strike but you have to do these things occasionally, you have to make a stance and demonstrate that things are not right to the wider public. (Myles, clinical)

In other instances, the psychosocial dynamics for some workers were influenced by the internal conflicts between staff, “I’ve never had a problem with a patient ever in my life, it’s just colleagues” (Ethan, clinical). This tension undermined the psychological safety HCWs expected in their work environment, and involved management, those of the same level and those trained in different areas, negatively affecting multidisciplinary-team working. A lack of psychological safety was related to feelings of being unable to speak up due to fearing how other colleagues would react, as one participant noted “in a top-down hierarchal organisation like the NHS, it’s hard to go against the grain” (Paul, non-clinical). Those working in mental health roles pointed to the irony of managing their own mental health. The absence of psychological safety led to distress, where HCWs highlighted their disappointment when the values of a mental health sector were not upheld by those working there.

It’s the surprise of the expectation of working within a therapy service that sometimes doesn’t think therapeutics and the injury that comes from other therapists. (Roza, clinical)

The psychosocial dynamics of the workplace was affected when there was an imbalance in reciprocity, which impacted HCWs’ mental strain. When someone failed to manage their responsibilities, it created an increased workload burden for others. This occurred when working with other services, as Jessica expressed her frustrations with general practitioners “who don’t pick up the slack in their workload that eases ours”. Helena described a growing feeling of isolation, attributed to a perceived generational divide in her team:

We were a very strong team. We all got on with each other, but when there’s no desire from the new members of the team to integrate and try their best it does form an issue. I feel like our team is probably a little divided in that we have the older members of the team who are probably preparing to kind of leave. (Helena, non-clinical)

### Theme 2: external context and the moral cost of culture

This theme captures the organisational culture of the NHS, and how this, as well as HCWs’ own psychological landscape/internal context, shaped their experiences of MI.

#### Organisational culture

This subtheme explores the organisational culture of the NHS, including opacity, (ie, where HCWs perceived those higher up in the Trust to lack transparency when it came to decisions and information sharing), blame and tick-box culture. Most staff felt those higher up in the Trust induced a false impression of the current state of the NHS, touching on the issue of hierarchy raised in the previous theme. They felt frustrated when management promoted their achievements and celebrated support only in the aftermath. This left staff believing the Trust was disingenuous in their offerings of support as the root cause of their issues were ignored:

Management are like, we’re so amazing, we’ve produced this and we’re supporting you in this. I shouldn’t need that in the first place to do my job, all you’re doing is bandaging a wound, you’re not preventing me from getting wounded in the first place. (Sarah, clinical)

A culture of opacity was highlighted through the HCWs’ lack of awareness of certain organisational processes and structures, for example, many were unaware how funding for the organisation operated. Not knowing the reasoning behind decisions left many feeling confused, perceiving decisions to be unfair or speculating about budgets. Failure to share information about plans for the organisation resulted in staff feeling unmotivated to try their best:

If I believe that we’re doing something amazing, then it motivates me, but I don’t really know what direction we’re going in, I don’t really understand all the whisperings, why would I put my heart and soul into this role. (Helena, non-clinical)

Additionally, employees felt the Trust could be more honest with new starters about their roles. Some HCWs showed sadness that their profession was not “spoken about in terms of enjoyment, pleasure or aspirations anymore” (Sarah, Psychoanalyst), and many believed the healthcare field to be unattractive to young people, with some highlighting the disillusion that students experienced. The opacity around systemic issues, and the false sense of success, may contribute to this.

Before they start, maybe some experienced staff going out and having that talk to them about the positives about working in the organisation, being realistic about it, or what tools they might need to hone. (Bronagh, non-clinical)

A culture of opacity was associated with the no blame policy. “They always say it’s a no blame culture, I don’t know if anybody fully believes” (Tobias, clinical). Roza, (clinical), noted that a blame culture resulted from focusing in on isolated facts and that a more holistic approach to managing mistakes is needed. The psychosocial dynamics and the lack of psychological safety experienced by HCWs also relates to this subtheme, where fear of punishment discouraged accountability. Indeed, blame culture emotionally burdened staff, causing them to internalise guilt, even if problems stemmed from organisational errors:

You’re sat in coroners court, you’re asked a question, and you relay why you didn’t do a certain thing or why you didn’t document a certain thing, and I’ve seen some staff, just that guilt has been absolutely tremendous, and maybe it might have been an organisational thing, but they take it very personally. (Bronagh, non-clinical)

Similarly to the frustration expressed when Trusts celebrated after-the-fact support, many felt their Trust’s focus was to meet metrics at the expense of genuine problem solving. Tick-box culture permeated multiple aspects of the NHS, with HCWs relating it to staff surveys, training, mental health checks and hours of supervision. Regarding the latter, Sarah recalled her seniors prioritising easy to measure metrics, rather than subjective meaningful outcomes.

Quality versus quantity, I think the NHS miss that completely. They just go, what’s the easiest thing to tick. (Sarah, clinical)

#### Sources of MI

The organisational culture relates to this subtheme, as tick-box culture was often driven by Trusts wanting to meet targets, consequently many HCWs experienced moral distress as their attention to patient care was redirected. Here we saw MI by acts of commission, with overlaps of omission. For example, Ethan (clinical) reported that they were told to ask patients to give feedback on the service, but in doing so, felt their job was ‘customer service based rather than patient centred’. Thus, the priorities of the business managers (reaching targets) and clinical staff (patient care) were misaligned.

A perceived lack of control also relates to this subtheme, as many HCWs felt they were unable to fulfil their duty of care towards patients due to reasons outside their control. Similarly, there were elements of omission as well as commission seen here. Resource shortages led to an over-reliance on telehealth, and this approach led to moral conflict in HCWs as they felt unable to offer additional interventions, for example, hospital-based electroencephalograms/X-rays. This conflict also occurred in mental health professionals, where they could only offer patients online therapy, despite patients wanting face-to-face therapy.

The lack of room space for therapy sessions might impact on the push to see more people online and that’s not OK because I think it’s better to do it based on client need rather than we don’t have enough rooms. (Roza, clinical)

HCWs described their initial betrayal caused by the mistakes made during the pandemic. This included the government’s symbolic gestures rather than tangible support, through the ‘clap for carers’ movement, as well as money wasted on inadequate PPE. Many also perceived the government to be reluctant to acknowledge their own mistakes and HCWs’ past efforts during the acute phase of COVID-19. In this way, the initial betrayal caused by the original mistakes turned into a secondary betrayal caused by a lack of recognition.

Clapping is not putting food on tables and paying bills with the high cost of living. But again, morally we all went in and faded away from our families. We all sacrificed in that way, but it’s not being recognised then and it hasn’t been recognised. (Alice, clinical)

This type of betrayal paired with government failures in the normalisation period, like unfair working conditions and pay, left many feeling hopeless about any future change.

I don’t think we are in a better position, if this happened again and we went into lockdown tomorrow, I can’t think of a single thing that has changed. (Helena, non-clinical)

### Theme 3: responses to PMIEs

This theme captures the ways in which HCWs responded to PMIEs, through emotional outbursts and questioning their sense of self or career, and adaptively, through seeking support, advocating for change and adopting positive mindsets.

#### Maladaptive responses

This subtheme encapsulates the negative responses to moral distress. The experiences discussed in all subthemes so far capture various PMIEs, and for many HCWs these led to responses such as anger, anxiety and fatigue. Alice (clinical) referred to her worry, which arose from the fear of being unable to perform all her duties. Despite caring for her patients, her relief at the end of her shift stemmed from an absence of negative outcomes rather than a feeling of accomplishment, noting ‘at the end of the day you feel, okay, well nothing adverse happened’. Regarding anger, Jessica’s trigger was the unprofessional communication from her colleague which manifested into an outburst of anger through shouting back:

I had an incident at work where a consultant shouted at me down the telephone, so I shouted back and I just thought this is not the way I operate, this is not who I am. (Jessica, clinical)

This reflects acts of commission, where HCWs acted in ways that violated who they wanted to be, acting in anger and adjusting their standards downwards, prioritising survival over success. In doing so, they questioned their sense of self. Other HCWs questioned their career. Mentioned previously, initial betrayal created in the acute stages of the pandemic and the secondary betrayal (lack of recognition) generated in the normalisation period, caused many HCWs to lose hope in the NHS, prompting them to search for other roles, leave their Trust or leave the NHS completely.

I’ve got to the point where I am looking for other jobs and other opportunities because I feel like I’ve banged my head against a brick wall for long enough and things aren’t changing. (Helena, non-clinical)

#### Adaptive responses

Some HCWs reacted adaptively to PMIEs through advocacy. This included speaking up for themselves, being consistent with providing feedback to their managers and showcasing their teams’ achievements. This strategy was, however, more common among those with supportive colleagues and those with greater responsibility and control:

I’ve got a little bit more advocacy for the team and I can speak up for them, whereas before in covid times it was like well this is what you’re going to do and you’re doing it. (George, clinical)

In contrast, other HCWs viewed solely voicing their frustrations as unhelpful and saw acceptance as a route to resolving moral distress.

That doesn’t mean accepting it means you approve it, but there’s no point just moaning about it. (Tobias, clinical)

Most HCWs who used advocacy as a strategy to manage their moral distress also demonstrated a positive mindset, where they recognised a reality where outcomes were out of their control, accepted uncertainty and recognised their limitations. Some HCWs focused on their role, concentrating on honing their skills instead of getting involved in other situations/people.

Those things will either happen or they won’t, I can’t control them, I think I’ve done what I can to protect myself from some of the other likely morally injurious things that could happen. (Joshua, clinical)

Another adaptive response that HCWs developed was to reframe a situation to reduce personal impact. Over half of HCWs recognised that their experiences were not unique to themselves. The normalisation of challenges helped them understand that failures were not personal failures but were part of wider systemic issues. When they perceived others to be going through a similar situation it promoted a shared struggle which reduced moral distress.

It’s nice to just learn and grow as people in this job when you have informal conversation, then you can overhear conversations as well and you think, yeah, that’s me, I’m glad It’s not just me. (Roza, clinical)

This adaptive strategy was facilitated by informal conversation, highlighted above. For Julia, (clinical) talking with her nursing friends was ‘in itself like counselling’, while others found formal counselling beneficial. Some engaged in formal help-seeking behaviours, but often only when the situation became critical.

I had no motivation to be at work. Felt everything was just sort of pointless so that prompted me to refer myself to Practitioner Health and then have some time away. (Joshua, clinical)

## Discussion

We explored how HCWs’ experiences of MI developed over 3 years by following up an established cohort of NHS staff last interviewed in late 2021 about their PMIEs during COVID-19. This study contributes to the MI literature by exploring MI during the postpandemic normalisation period, which is quite a different context socially, politically and globally than during the pandemic. We identified a complex interplay between the internal (individual) and external (organisational/societal) contexts HCWs faced, along with adaptive and maladaptive responses. Our findings suggest that there is a pressing need for integrated strategies to enhance organisational structures that can help staff to feel supported by their employer.

An overarching finding was that participants tended to discuss morally injurious experiences in quite general terms, rather than focusing on detailed examples or very extreme examples. The examples given tended to be of ‘everyday’ experiences, for example, witnessing patients being on treatments that are not appropriate (omission), staff going on strike (commission), lack of support from senior staff and lack of appropriate rooms/resources (betrayal). There was a sense of what could be termed ‘diffuse moral injury’, with ‘the little things’ adding up to a pervasive sense of participants’ values being at odds with those of their organisations.

Three main themes and six subthemes were identified. The first theme concerned the ‘internal context’ and the psychological landscape of HCWs and how ‘psychosocial dynamics’ and ‘control’ impacted their well-being/motivation. Consistent with previous research,^20^ a lack of psychological safety (eg, trust in colleagues, managers and the wider system) hindered open dialogue. Similarly, our baseline interviews^7^ found that many HCWs considered managers unapproachable. This issue is also reflected in the NHS staff surveys, where the percentage of staff feeling unsafe to voice concerns in 2021 (38%) remained unchanged in 2023.^21^ Feeling undervalued and misunderstood appeared to stem from a misalignment between staff members’ expectations and reality. This was apparent for staff whose expectations of psychological safety were unmet and, as they perceived it, patients who felt their issues to be urgent and were frustrated by what they appeared to see as lack of response from HCWs. Since our 2021 interviews,^7^ patient behaviour seems marked by increased health anxiety,^22^ likely exacerbated by worsening waiting times.^23^

Job control was more salient in this study than identified previously.^7^ Insufficient control was linked to workforce pressures, echoing long established research showing that employees facing high demands and low control often exhibit stress/burnout.^24^ HCWs felt powerless over recruitment decisions and constrained by resource shortages which impaired their ability to deliver optimal care. These pressures were also felt during the pandemic, but their continuation appears to have added to feelings of betrayal. In the wider occupational health literature, job control is recognised as a contributor to work-related stress and poorer occupational outcomes.^25^

The second theme concerned the ‘External context’ and its influence on ‘organisational culture’. Previous research found HCWs believed employers to be dishonest with the public about hospital conditions and PPE sufficiency.^9^ Similarly, many of our participants felt heads of departments misrepresented the success of the NHS since the pandemic to staff, creating a culture of opacity. While this specific issue did not arise in the 2021 interviews, a tick-box culture did and this issue has persisted. A culture of blame, echoing previously identified issues of self-blame,^7^ suggests blame may manifest both internally and externally, contributing to guilt and distress.

‘Sources of MI’ during the pandemic’s acute stages seemed to have evolved. Initial feelings of betrayal over lax government restrictions^7 8^ have developed over time into a secondary betrayal due to the perception of the government’s continued lack of accountability for their own mistakes and of acknowledgement of the sacrifices made by HCWs. PMIEs related to telehealth persist.^7^ Initially introduced to provide remote consultation and reduce infection risk,^26^ telehealth is now used to cut waiting times. Some HCWs felt this constrained their ability to offer adequate care, reinforcing the persistence of PMIEs beyond crisis points. Whereas MI was once linked to sudden, high-stake situations,^7^ it has since been dominated by systemic dysfunction.

Where PMIEs cannot be prevented, reconciliation appears essential. ‘Moral repair’ interventions may help reconcile moral violations, strengthening relationships and belief systems.^1^ Similar to our 2021 findings, informal conversations reportedly reduced moral distress by allowing HCWs to reframe their experiences and recognise their challenges as systemic rather than individual failings.

However, not all HCWs responded adaptively, and some HCWs expressed anger and intentions to leave the NHS. Research suggests moral repair is a dyadic process, with non-engagement being a key barrier to restoring relationships.^27^ Future interventions should aim to channel this anger constructively, fostering engagement and healing. At the time of these interviews, England remained under the same Conservative government as during COVID-19’s onset. Future research could explore how HCW attitudes shift under the new administration elected in July 2024, particularly in relation to trust and the concept of secondary betrayal.^8^

Overall, in following up HCWs 3 years, we found that, while many PMIEs remain (eg, blame culture, lack of resources, constrained care), the period since the pandemic has seen MI compounded via secondary betrayal, with lack of autonomy and control playing a larger role.

### Implications

Our findings should be considered in the context of the fact that we achieved follow-up participation of only 50%. It is possible that non-responders left their Trust or the NHS, as several email invitations bounced back, although this could have been due to other reasons (eg, name changes). As a result of this, our sample reflects only those still working, which may underestimate the true extent of MI in the NHS, and the adaptive responses identified may be over-represented. This may limit the transferability of the findings. Future research could purposefully recruit individuals who have already left their roles, to capture insights into the impact of MI on workforce retention and well-being. Another limitation is that, while some participants reported experiencing PMIEs, they sometimes struggled to provide detailed examples. While probing mitigated this to some extent, future research could encourage reflection before interviews to support richer responses, for example, by using storytelling or diary techniques.

With those points in mind, this analysis highlights the interconnection between HCWs’ internal and external contexts. To improve psychosocial dynamics*,* Trusts could provide development training on constructive criticism, setting expectations and psychological safety. Clarifying roles and encouraging open dialogue may reduce internal conflict.^28^ Implementing systems to provide patients with real-time updates and reasons for delays may ease pressures on staff. To address insufficient control, Trusts could revise decision-making protocols to include wider HCW input. Expanding the hours of occupational health services could improve access to support.

Improving discussion and transparency may combat a culture of opacity.^29^ However, leaders must balance openness with the risk of overwhelming staff.^30^ Emphasising learning over blame and identifying causes of issues may help reduce recurrence of errors and associated guilt.^31^ Senior leaders could act as ‘champions’ for initiatives, and ‘bite sized’ training formats may increase uptake and reduce overwhelm. To address betrayal-based MI, Trusts could trial moral repair interventions, especially compassion-focused interventions. However, more evidence is needed to determine which approaches are most effective.

## Conclusions

Our findings help to fill a gap in the literature by exploring the complexity of MI among HCWs in the postpandemic normalisation period. This study highlights how individual experiences are shaped by broader organisational culture and perceived betrayals. Future research should continue to explore the development and application of moral repair-based interventions, particularly in response to systemic or political betrayal.

## Supplementary material

10.1136/bmjopen-2025-115402 online supplemental file 1

## Data Availability

Data are available upon reasonable request.
