# Supplementary material for: NHS healthcare worker experiences of moral injury: a follow-up qualitative study in England using reflexive thematic analysis
Source: BMJ Open. 2026 Aug 18;16(8):e115402. doi: 10.1136/bmjopen-2025-115402 (PMC13504529; doi:10.1136/bmjopen-2025-115402)
Supplement: Supplementary file 1 [file bmjopen-2025-115402.reviewer_comments.pdf]

PEER REVIEW HISTORY

BMJ Open publishes all reviews undertaken for accepted manuscripts. Reviewers are asked to complete a checklist review form and are provided with free text boxes to elaborate on their assessment. These free text comments are reproduced below.

ARTICLE DETAILS

Title (Provisional)

NHS healthcare worker experiences of moral injury: A follow up qualitative study in England using reflexive thematic analysis

Authors

Higginbottom, Erin; Stoeck, Friederike; Stevelink, Sharon; Hegarty, Siobhan; Raine, Rosalind; Rafferty, Anne Marie; Greenberg , N; Wessely, Simon; Rahman, Hassan; Lamb, Danielle

---

VERSION 1 - REVIEW

**Reviewer**	**1**
**Name**	**Ward, John**
**Affiliation**	**University of Exeter**
**Date**	**12-Feb-2026**
**COI**	**None**

Thank you for submitting this interesting manuscript on the nature of moral injury post pandemic. Overall I think this is a good paper worthy of publication, however I have a few minor points to put to the authors that I think would refine the paper.

Strengths and Limitations; I think you over-interpret what emails bouncing means re staff leaving the NHS. NHS emails close down all the time (as I'm sure you all appreciate) for a huge variety of reasons.

As you say in your Strengths and Limitations--it does feel that discrete MIs are harder to find for your participants, and rather they are reporting 'diffuse moral injury' at times. It would be nice to see this more reflected in your discussion--I can't tell whether or not you you as the authors expected or didn't expect the vague themes in the results (as a reader, this not being addressed makes me feel that I don't understand moral injury at all!)

I also think I am struggling in reading this paper slightly with the idea of comparing one set of moral injuries in a unique global, societal, political context with a new context (and a context from 2024 from before the last general election--which is different to the context I read the paper in now). However, I can't see this reflected in your manuscript.

Background:

I think slightly more expansion about what moral injury is would be useful. You say it is not a clinically diagnosable condition--I don't think anyone would think that it is? Perhaps more exploration of the wider moral injury literature in a broader sense (what is a moral injury in practice c.f. the narrow definition, or in a pandemic-world) would be helpful. The pandemic feels like such a specific context to put our understanding of MI in for reading the paper that the rest of the paper feels slightly vague re moral injury by contrast.

Methods:

I think your occupations are too specific--I'd be worried that participants could be identified (e.g. the one doctor, who later you tell me referred himself to Practitioner Health). I would consider some further aggregation of roles (unless there is a very specific reason why you think specifying people's exact job titles is useful beyond, for example, 'registered healthcare professional').

Some more description as to exactly what is in the topic guide is also needed in your methods, otherwise it makes your results very hard to understand.

Results:

Again, you also use some job titles not reflected above--including Sarah, a psychoanalyst, (which is not in Table 1 as a profession...) and Tobias, AMHP.

Discussion:

I think there is a big issue with the discussion presenting the 2021 data as a comparator for this data. I don't think that qualitative findings (and particularly in two populations drawn from the same study, but different populations) can be compared 'like for like' in this quasi-quantitative way. I don't take issue with the points made in the discussion, but I think more reflection on what the participants tell us about the nature of change to MI over time is what the discussion should focus on--there appears to be an over-reliance in parts on 'the 2021 paper said X, this time we found Y'. For example-"job control was more significant"--on what basis? Your paragraph at the end of page 19 (lines 42-56) is a better example of how you have interpreted your findings about change from this series of interviews in their own right.

**Reviewer**	**2**
**Name**	**Gupta, Snehil**
**Affiliation**	**AIIMS Bhopal, Department of Psychiatry**
**Date**	**14-Mar-2026**
**COI**	**None**

Dear Authors,

I commend your work, particularly the topic which was apt though can be controversial as well.

My comments can be found in the attached file.

Regards, Reviewer

**** **The reviewer provided a marked copy with additional comments. Please contact the publisher for full details.** ****

**Reviewer**	**3**
**Name**	**Colville, Gillian**
**Affiliation**	**St George's Hospital**
**Date**	**18-Mar-2026**
**COI**	**None**

This paper describes the results of a qualitative interview study with a group of NHS staff previously interviewed about their experience of moral injury in the course of their work during the pandemic.

The continuing impact of the pandemic on NHS staff is an interesting topic and the Methods are clear and appropriate.

Those interviewed reported their original experience of moral injury had been further compounded by ongoing moral injury related to continuing staffing issues; the perceived lack of government accountability for failings during the pandemic and lack of appreciation of what they had been through.

Interestingly some felt there was an over reliance on tele health since the pandemic and questioned whether this had improved quality of care.

The findings are however presented in quite abstract terms. As the authors themselves acknowledge, interviewees reported experiencing morally injurious events but could not give examples. Staff interviewed sound fed up in their jobs but it is a bit unclear why or whether this is primarily because of moral injury or how their feelings have developed over time in the 3 years since they were last interviewed. These results read more like a description of a general malaise in the NHS, due to chronic lack of resources, than a specifically pandemic related phenomenon.

Weaknesses include the fact that no hypotheses are given, the n was 15 (all of whom were White and only one of whom was a doctor) out of an eligible group of 30 and it is not clear how representative this final group was. Given the well documented link between moral distress and intention to leave a job, it may be that the reason it was not possible to contact half the original qualitative sample is that they left their jobs because of their moral

distress/injury. This may have been ‘adaptive’ at an individual level rather than mal-adaptive?

MAIN POINTS

There is insufficient information about the larger sample from which this sample is drawn. Please provide more information on the quantitative CHECK study and on how the people chosen for qualitative interviews in 2021 were chosen. Reference to the CHECK study is provided but only to the protocol not to any results.

‘Moral injury’ is the term used in this paper but the themes from the earlier qualitative study (provided in the suppt) use the term ‘moral distress’. Grateful for an explanation as to what, if any , difference the authors think there is between these terms.

Did anyone talk about using whistleblowing processes in their NHS Trust?

The interview questions are quite leading – they seem to prompt the idea of betrayal. The authors do acknowledge in the Strengths and Limitations section that this wording may have biased their results.

Was there a particular reason for repeating interviews 3 years later with this group?

Were the main CHECK sample also followed up in relation to the quantitative findings?

What sort of interventions do the authors think are feasible or would help? (apart from structural solutions such as increased staffing/resources). Did any interviewee give an example of any useful intervention?

What is justification for focussing on MI in healthcare workers after the pandemic as opposed to depression or PTSD (ie diagnosable, treatable conditions) – or is there more data on this from the CHECK study published elsewhere?

It is important to note that the experience of a PMIE is quite common in healthcare and does not necessarily lead to MI (see recent research using the MIDS). Could the fact that the interviewees struggled to come up with examples may be because they were not still troubled by them?

The authors acknowledge (p5) that these findings cannot truly demonstrate a ‘trajectory’, other than simply comparing concerns just after the worst of the pandemic (2021) and those in the longer aftermath (2024), suggesting in the Introduction that more longitudinal data is needed (p5). What then is the justification for this study of only 15 people?

Important to acknowledge pre-pandemic rates of depression, PTSD, moral distress.

MINOR POINTS

Abstract: in Conclusion suggest ‘emphasising the need for interventions’ rather than ‘emphasising the importance of interventions’

P10 suggest 3. ‘Responses to PMIEs ‘rather than ‘Responses of PMIEs’

P11 typo 1i should be 1ii

VERSION 1 - AUTHOR RESPONSE

**Reviewer: 1**	
Thank you for submitting this interesting manuscript on the nature of moral injury post pandemic. Overall I think this is a good paper worthy of publication, however I have a few minor points to put to the authors that I think would refine the paper.	We thank the reviewer for their kind comments.
Strengths and Limitations; I think you over-interpret what emails bouncing means re staff leaving the NHS. NHS emails close down all the time (as I'm sure you all appreciate) for a huge variety of reasons.	We have removed this sentence from the limitations section (page 3).
As you say in your Strengths and Limitations--it does feel that discrete MIs are harder to find for your participants, and rather they are reporting 'diffuse moral injury' at times. It would be nice to see this more reflected in your discussion--I can't tell whether or not you you as the authors expected or didn't expect the vague themes in the results (as a reader, this not being addressed makes me feel that I don't understand moral injury at all!)	We agree that participants did talk in more general terms rather than list specific, extreme, morally injurious experiences. However, we believe there are discrete examples of potentially morally injurious experiences given by participants, e.g. witnessing patients being on treatments that are not appropriate (omission), staff going on strike (commission), lack of support from senior staff and lack of appropriate rooms/resources (betrayal). We have added a paragraph at the start of the Discussion to address this (page 20).
I also think I am struggling in reading this paper slightly with the idea of comparing one set of moral injuries in a unique global, societal, political context with a new context (and a context from 2024 from before the last general election--which is different to the context I read the paper in now). However, I can't see this reflected in your manuscript.	We believe we have addressed the shift in political context (e.g. "Future research could explore how HCW attitudes shift under the new administration elected in July 2024" page 22; and the penultimate paragraph of page 21, which discusses perceptions of the government's continued lack of accountability over time). However, we take the point that this could be made more explicit elsewhere as well, so have added points on this in the first paragraph of page 20 and page 21.
Background:<br>I think slightly more expansion about what moral injury is would be useful. You say it is not a clinically diagnosable condition--I don't think anyone would think that it is? Perhaps more exploration of the wider moral injury literature in a broader sense (what is a moral injury in practice c.f. the narrow definition, or in a pandemic-world) would be helpful. The	We thank the reviewer for these helpful comments. We propose retaining the point about MI not being clinically diagnosable, as those new to MI research may not be aware of this point. We have added additional explanation and examples on page 5.

pandemic feels like such a specific context to put our understanding of MI in for reading the paper that the rest of the paper feels slightly vague re moral injury by contrast.	
Methods:<br>I think your occupations are too specific--I'd be worried that participants could be identified (e.g. the one doctor, who later you tell me referred himself to Practitioner Health). I would consider some further aggregation of roles (unless there is a very specific reason why you think specifying people's exact job titles is useful beyond, for example, 'registered healthcare professional').<br>Some more description as to exactly what is in the topic guide is also needed in your methods, otherwise it makes your results very hard to understand.	We have amended the occupation data simply to the broad roles of clinical/non-clinical.
Results:<br>Again, you also use some job titles not reflected above--including Sarah, a psychoanalyst, (which is not in Table 1 as a profession…) and Tobias, AMHP.	We have updated these job titles to clinical and non-clinical.
Discussion:<br>I think there is a big issue with the discussion presenting the 2021 data as a comparator for this data. I don't think that qualitative findings (and particularly in two populations drawn from the same study, but different populations) can be compared 'like for like' in this quasi-quantitative way. I don't take issue with the points made in the discussion, but I think more reflection on what the participants tell us about the nature of change to MI over time is what the discussion should focus on--there appears to be an over-reliance in parts on 'the 2021 paper said X, this time we found Y'. For example-"job control was more significant"--on what basis? Your paragraph at the end of page 19 (lines 42-56) is a better example of how you have interpreted your findings about change from this series of interviews in their own right.	For clarity, the population of the current study is drawn from the participants from the 2021 study. All previous interview participants were invited to be interviewed again, with 15 taking up the offer, so the current paper and 2021 paper report on interviews from the same participants (excluding those who declined to participate in this round of interviews). We did not aim to compare findings directly between individual participants' responses from the 2021 and 2024 (this would have been an interesting design, but not what we did), but rather to consider how experiences and different themes had developed over time. We take the reviewer's point about the term 'significant', and have amended this to 'salient', which we feel does represent the interpretation we made of the data.
Reviewer: 2	
I commend your work, particularly the topic which was apt though can be controversial as well.	We thank the reviewer for their kind comments.

Abstract -Background- highlight research gap in the background	We believe we have done this, on page 7: "Given the limited available research into MI among HCWs in the post pandemic period, further investigation is needed to understand the development of MI experienced by HCWs. Better understanding of MI in HCWs will identify opportunities for prevention (where possible) and support to be provided where necessary."
Limitation - authors have mention the role of "encourage reflection"- they may also suggest ways to do that, emotion chart, cognitive continuum, story telling, etc.	We have added this helpful suggestion.
Introduction - some context must be provided in the introduction about what administrative or functional changes/amendments were brought by the NHS/trust with regard to clinical care and HCW's wellbeing through appropriate citations. This is make the findings relevant to wider audience, otherwise, this paper will have a very limited readership	We are not quite clear what this point is suggesting – does the reviewer mean that we should provide context about changes made between the pandemic and post-pandemic periods? In response to Reviewer 1's comments, we have added a paragraph at the start of the Introduction (page 5) outlining the different types of morally injurious experiences over the two periods, and hope that this will suffice to address this point.
Participants: (3) reported<br>that they had experienced a PMIE through the 9-item MI events scale. How the PMIE or MI was established by the researchers? was it only based on subjective reporting. What about the participation bias, only those HCWs consented to participate in the qualitative study who have MI experiences or PMIEs?	This is correct, the MIES is a self-report scale, and we have added clarity about this in the Participants section on page 8. The reviewer is also correct that only those who reported having experienced a PMIE were invited to interview, as outlined in point (3) of the Participants section on page 8.
Data collection/qualitative interview approach - any specific reason why did authors not conduct FGDs, which otherwise could have enriched the data collection?	The rationale for conducting individual interviews rather than focus groups was to enable participants to speak more freely about their experiences than they might have felt able to in a group discussion.
RTA- The reflexive journal was helpful in noting our own…? authors can cite some examples here.	We have added an example in the Reflexivity section on page 10.
Reviewer: 3	
This paper describes the results of a qualitative interview study with a group of NHS staff previously interviewed about their experience of moral injury in the course of their work during the pandemic.<br><br>The continuing impact of the pandemic on NHS staff is an interesting topic and the Methods are clear and appropriate.	We thank the reviewer for this helpful summary.

Those interviewed reported their original experience of moral injury had been further compounded by ongoing moral injury related to continuing staffing issues; the perceived lack of government accountability for failings during the pandemic and lack of appreciation of what they had been through.<br><br>Interestingly some felt there was an over reliance on tele health since the pandemic and questioned whether this had improved quality of care.	
The findings are however presented in quite abstract terms. As the authors themselves acknowledge, interviewees reported experiencing morally injurious events but could not give examples. Staff interviewed sound fed up in their jobs but it is a bit unclear why or whether this is primarily because of moral injury or how their feelings have developed over time in the 3 years since they were last interviewed. These results read more like a description of a general malaise in the NHS, due to chronic lack of resources, than a specifically pandemic related phenomenon.	As above, participants did give examples, but talked about their experiences in more general terms. We agree that the results indicate a pervasive malaise, and that this is not specifically a pandemic related phenomenon – the aim of the paper was to explore experiences since the pandemic. We believe we have addressed this point above, in relation to comments from Reviewer 1.
Weaknesses include the fact that no hypotheses are given, the n was 15 (all of whom were White and only one of whom was a doctor) out of an eligible group of 30 and it is not clear how representative this final group was. Given the well documented link between moral distress and intention to leave a job, it may be that the reason it was not possible to contact half the original qualitative sample is that they left their jobs because of their moral distress/injury. This may have been 'adaptive' at an individual level rather than mal-adaptive?	It is not usual to give hypotheses in qualitative research, as we did not approach the data with fixed ideas about what it would or wouldn't show, but rather with openness and curiosity about the experiences participants shared with us. Similarly, qualitative research does not aim to be representative, but for findings to be transferrable. We agree with the point about intention to leave sometimes being adaptive, and had addressed this point in the reflexivity section on page 10.
There is insufficient information about the larger sample from which this sample is drawn. Please provide more information on the quantitative CHECK study and on how the people chosen for qualitative interviews in 2021 were chosen. Reference to the CHECK study is provided but only to the protocol not to any results.	We have added a reference to a pre-print of a paper that provides information on the full NHS CHECK cohort (this paper has been submitted but is not yet published).
'Moral injury' is the term used in this paper but the themes from the earlier qualitative study (provided in the suppt) use the term	Moral distress refers to the psychological discomfort that arises when someone feels that they should act in accordance with their

'moral distress'. Grateful for an explanation as to what, if any , difference the authors think there is between these terms.	own values, but is prevented from doing so by external constraints (e.g. policy, hierarchy, lack of resources). It is usually situational and potentially reversible if constraints change.<br>Moral injury refers to a deeper, enduring harm to one's moral identity that results from participating in, witnessing, or being unable to prevent acts that violate deeply held moral values. It involves lasting guilt, shame, betrayal, or loss of trust and can persist long after the situation has ended.<br><br>We have added this information to the introduction on page 5.
Did anyone talk about using whistleblowing processes in their NHS Trust?	This is an interesting point, but none of the participants in this study discussed this issue.
The interview questions are quite leading – they seem to prompt the idea of betrayal. The authors do acknowledge in the Strengths and Limitations section that this wording may have biased their results.	As the reviewer notes, this point has been addressed.
Was there a particular reason for repeating interviews 3 years later with this group?	The rationale is that given at the end of the Introduction, to explore changes in experiences.
Were the main CHECK sample also followed up in relation to the quantitative findings?	They were, and there are quantitative papers forthcoming on this issue.
What sort of interventions do the authors think are feasible or would help? (apart from structural solutions such as increased staffing/resources). Did any interviewee give an example of any useful intervention?	No participants gave concrete examples of specific interventions. We agree with the reviewer that structural solutions would be helpful, such as increased staffing and resources. In addition, we believe the findings support the idea that greater job control and interventions to increase team cohesion and organisational culture may help to reduce morally injurious events.
What is justification for focussing on MI in healthcare workers after the pandemic as opposed to depression or PTSD (ie diagnosable, treatable conditions) – or is there more data on this from the CHECK study published elsewhere?	There is indeed other data from NHS CHECK on the topic of depression and PTSD.
It is important to note that the experience of a PMIE is quite common in healthcare and does not necessarily lead to MI (see recent research using the MIDS). Could the fact that the interviewees struggled to come up with examples may be because they were not still troubled by them?	We agree with the reviewer that this is an important point. We have added information on this in the Introduction on page 5.

The authors acknowledge (p5) that these findings cannot truly demonstrate a 'trajectory', other than simply comparing concerns just after the worst of the pandemic (2021) and those in the longer aftermath (2024), suggesting in the Introduction that more longitudinal data is needed (p5). What then is the justification for this study of only 15 people?	The purpose of qualitative research is not to analyse data from large representative groups, but to explore in depth the experiences of individuals. It is still relatively unusual in qualitative research to carry out interviews with the same participants, despite the insights such longer term work can provide, hence the novelty of this study.
Important to acknowledge pre-pandemic rates of depression, PTSD, moral distress.	We believe this has been addressed in the Introduction.
Abstract: in Conclusion suggest 'emphasising the need for interventions' rather than 'emphasising the importance of interventions'	We have made this edit.
P10 suggest 3. 'Responses to PMIEs 'rather than 'Responses of PMIEs'	We have made this edit.
P11 typo 1i should be 1ii	We have made this edit.

VERSION 2 - REVIEW

Reviewer 1
Name Ward, John
Affiliation University of Exeter
Date 30-Jul-2026
COI

Thanks to the authors for their thoughtful revisions to this manuscript. I think better context in the introduction is helpful and it is nice that the authors have added an implications paragraph in response to feedback.

I still maintain my point about 'MI not being clinically diagnosable'. I get that this might be unfamiliar ground, however I can imagine at least to doctors it reads as a bit of a moot point and therefore one might be tempted to gloss over the rest of the sentence (which should be read, as it makes a very important point). Perhaps leading with the important bit here about its association with MI might be good (and then the more debatable/unclear bit of the sentence the reader can take or leave, rather than disregarding the whole sentence).

I'm also personally unsure about comparing the 2021 and 2024 studies rather than interpreting these findings in their own right in the discussion, even if they are drawn from the same population, but I think that's a matter of personal opinion.

VERSION 2 - AUTHOR RESPONSE

Reviewer: 1
Dr. John Ward, University of Exeter
Comments to the Author:
Thanks to the authors for their thoughtful revisions to this manuscript. I think better context in the introduction is helpful and it is nice that the authors have added an implications paragraph in response to feedback.

I still maintain my point about 'MI not being clinically diagnosable'. I get that this might be unfamiliar ground, however I can imagine at least to doctors it reads as a bit of a moot point and therefore one might be tempted to gloss over the rest of the sentence (which should be read, as it makes a very important point). Perhaps leading with the important bit here about its association with MI might be good (and then the more debatable/unclear bit of the sentence the reader can take or leave, rather than disregarding the whole sentence).

We take the reviewer's helpful point, and have amended the sentence: "MI is associated with strong emotions such as guilt, anger, or shame, which may lead to mental disorders such as post-traumatic stress disorder, depression, or self-harm or suicidal behaviours3, but is not itself a clinically diagnosable condition."

I'm also personally unsure about comparing the 2021 and 2024 studies rather than interpreting these findings in their own right in the discussion, even if they are drawn from the same population, but I think that's a matter of personal opinion.
