# Supplementary material for: NHS healthcare worker experiences of moral injury: a follow-up qualitative study in England using reflexive thematic analysis
Source: BMJ Open. 2026 Aug 18;16(8):e115402. doi: 10.1136/bmjopen-2025-115402 (PMC13504529; doi:10.1136/bmjopen-2025-115402)
Supplement: Supplementary file 2 [file bmjopen-16-8-s001.docx]

# Supplementary Files

## Supplementary File 1

### Interview guide

#### Introduction

This interview is about your exposure to potentially morally injurious events in the workplace since your last interview at the end of 2021. Moral injury refers to the distress people experience, often in the form of shame, anger, guilt or disgust, when they see or carry out acts that clash with their deeply held moral or ethical beliefs and values. This may be a result of things you, or others, have done; things you or others did not do but you think should have happened; or from experiencing betrayal by those who you trusted or who should have been looking out for you.

Some examples of moral injury that have been recorded by NHS frontline staff during the pandemic include having to choose which of several sick COVID patients to ventilate due to resource constraints; not having the right medicines or equipment to save a person; putting non-COVID patients, or staff, at risk of contracting the virus due to inadequate PPE. Even after the pandemic moral injury can occur. For example, when understaffing leads to situations where you may provide insufficient patient care or when the waiting list is too long to properly divide your time between patients. You may feel like you are let down and unsupported by the NHS, government, or wider society. Of course, those are just examples, and your experience may be quite different. Does that all make sense?

Please do let me know if you have any questions at any point. The interview will last approximately 45 minutes and will ask you about your experience of events that potentially contribute to moral injury and the impact these events have had on you. I appreciate this may be a difficult topic to speak about. If at any point you’d like a break or to stop the interview we can pause and have a chat about how you’re feeling, reschedule to continue at another time, or stop completely. That being said, are you happy to begin the interview?

1. To begin, have there been any changes in your role or job since your last interview at the end of 2021?

- If you had a career switch, what were the reasons for this?

- Who made the decision regarding the change in your role (was this decision made for you, did you decide/agree to it?)

- [If there was a change] how did you feel about the change in role?

2. Can you please tell me about how you felt about working for the NHS since the pandemic and since your last interview?

- has this changed over time? If so, how and why?

3. Thinking about your work since your last interview, can you tell me about any experiences you’ve had, witnessed, or learnt about, that clashed with your moral or ethical values ?

- If they did, were these events a result of actions of you, or others? Please tell me a little about them?

- Did you experience a sense of betrayal from people you trusted, or who should have been looking out for since the pandemic/since your last interview, if so can you describe this briefly? (prompt for colleagues, management, organisation, healthcare staff you consulted, friends, family, government etc)

- On what occasions did you experience moral injury/events mentioned? (prompt: how frequent?)

- Roughly when did you experience the moral injury/injuries you described? (How was this similar to/different from the experiences you told us about in your original interview?

4. What impact did this/these morally challenging events have on you?

- Emotional (anger, guilt, shame, disgust, sadness, etc)

- Psychological (i.e. have you felt sad, depressed, burned out, anxious because of it? Have you felt at all on edge since it happened; had any unpleasant dreams/disrupted sleep since then; have you

- Found yourself avoiding anything that reminds you of it?)

- Relational (impact on family life, social life, colleagues, teamwork)

- Occupational (impact on ability to work, intention to change role/Trust/leave healthcare altogether, sick leave, team morale, feeling supported at work)

- Spiritual (impact on their faith/world view or beliefs)

- Functional (ability to do daily tasks at work and personal life)

- Other

5. How long have these feelings/effects lasted?

- Do you still feel that way (constant vs come and go)

- How strong are the feelings you describe?

6. Considering your recent experiences compared to your experiences when you were last interviewed in 2021, do you feel there are any differences?

- Are they more negative or positive or the same?

7. Have you experienced any long-term effects of the morally challenging events you experienced during the initial stages of the pandemic?

- Clarify if necessary – long term effects of moral injury reported in the first interview.

- If so, what have these been?

- How long have they affected you?

8. Is there anything that has been helpful, or unhelpful, in coping with these morally challenging experiences?

- Formal (counselling/issues with counselling, time away from work/not enough time away from work, access to support/ not enough access to support)

- Informal (friends, family, personality factors, resilience, coping strategies)

- Why have they been helpful/unhelpful?

9. Has the support you found helpful/unhelpful changed since your last interview at the end of 2021?

- If so, how?

10. Since your last interview, has your Trust done anything that makes dealing with exposure to potentially morally injurious events easier?

- Has your Trust done anything that makes dealing with exposure to potentially morally injurious events more difficult?

11. Going forward, what measures could be taken by your Trust to prevent or limit NHS staff experiences of potentially morally injurious events?

- Any measures that you think might be helpful in future crises?

- Any measures needed in general working life?

- Systemic changes within the NHS (e.g additional training , added regulations, inputting reporting system in the aftermath of PMIEs)

12. Going forward, what do you think could be done differently to help NHS staff cope with the experience of moral injury, if it can’t be avoided?

- Why/how would these things help?

- Are you worried about anything that might be a morally challenging experience for you to deal with in the future as we emerge from the pandemic? If so, can you tell me a bit about this?

- What impact might the changes you suggested have on your mental health, responding to future PMIEs

Interview Ending

Is there anything we haven’t yet covered that you feel is important to tell me?

How did you find the interview?

Thank you for talking to me today. I really appreciate your time. The experiences you’ve shared today will be really helpful to understand the effects that moral injury can have on mental health. We hope this information will inform Trusts on how they can best support staff like you and your colleagues. If you would like, we can email you later in the year to update you on our progress and tell you about the key findings from our study.

## Supplementary File 2

### Interview guide from the original 2021 study

#### Introduction

This interview is about your exposure to potentially morally injurious events in the workplace while responding to the COVID-19 pandemic. Moral injury refers to the distress people experience, often in the form of shame, anger, guilt or disgust, when they see or carry out acts that clash with their deeply held moral or ethical beliefs and values. This may be a result of things you, or others, have done; things you or others did not do but you think should have happened; or from experiencing betrayal by those who you trusted or who should have been looking out for you. Some examples of moral injury that have been recorded by NHS frontline staff to date include having to choose which of several sick COVID patients to ventilate due to resource constraints; not having the right medicines or equipment to save a person; putting non-COVID patients, or staff, at risk of contracting the virus due to inadequate PPE; feeling let down and unsupported by the NHS, government, or wider society. Of course, those are just examples, and your experience may be quite different. Does that all make sense? Please do let me know if you have any questions at any point. The interview will last approximately 45 min and will ask you about your experience of events that potentially contribute to moral injury and the impact these events have had on you. I appreciate this may be a difficult topic to speak about. If at any point you’d like a break or to stop the interview we can pause and have a chat about how you’re feeling, reschedule to continue at another time, or stop completely. That being said, are you happy to begin the interview? Could you please let me know your age

1. To begin, could you please tell me about your role(s) in the NHS during the pandemic?

- Has the nature of your job changed from what you were doing prior to the pandemic (e.g. redeployment).
- Who made the decision regarding the change in your role (was this decision made for you, did you decide/agree to it?)
- [If there was a change] How did you feel about the change in role?

2. Can you please tell me about how you felt working for the NHS before the pandemic?

- Has this has changed over the course of the pandemic and if so how and why?
- Have your relationships with colleagues/managers changed much since the pandemic?
- What about your relationship with people you are close to outside of the NHS like friends and family?

3. Thinking about your work during the pandemic, can you tell me about any experiences you had, witnessed, or learnt about, that clashed with your moral or ethical values(?).

- If they did, were these events a result of actions of you, or others? Please tell me a little about them?
- Did you experience a sense of betrayal from people you trusted, or who should have been looking out for you, during the pandemic, if so can you describe this briefly? (prompt for colleagues, management, organisation, healthcare staff you consulted, friends, family etc)
- On what occasions did you experience moral injury/events mentioned? (prompt: how frequent?)
- Roughly when did you experience the moral injury/ injuries you described? (How was this similar to/different from your experience before the pandemic?)

4. What impact did this/these morally challenging events have on you?

- Emotional (anger, guilt, shame, disgust, sadness, etc.)

- Psychological (i.e. have you felt sad, depressed, burned out, anxious because of it? Have you felt at all on edge since it happened; had any unpleasant dreams/disrupted sleep since then; have you found yourself avoiding anything that reminds you of it?)

- Relational (impact on family life, social life, colleagues, team work)

- Occupational (impact on ability to work, intention to change role/Trust/leave healthcare altogether, sick leave, team morale, feeling supported at work)

- Spiritual (impact on their faith/world view or beliefs)

- Functional (ability to do daily tasks at work and personal life)

5. How long have these feelings/effects lasted?

- Do you still feel that way (constant vs come and go)

- How strong are the feelings you described?

6. Have the experiences you described influenced other areas of your life/life stressors (e.g. difficulties at home)

- Has the pandemic created a backlog of work for you, and if so, has this impacted your ability to deal with the experiences you described?

- How have the morally challenging experiences you described changed your relationships to patients, if at all (more conflict, avoidance, less contact

7. What supports have you found helpful in coping with these morally challenging experiences?

-Informal (family friends, colleagues)

-Formal (psychological counselling, debriefing, time away from work etc.)

- Personal (coping strategies, resilience, personality factors etc.)

- Why have these things helped?

- How effective have these things been for you?

- Is there anything you think would be helpful to you right now?

8. What supports have you found unhelpful in coping with these morally challenging experiences?

- Informal (unsupportive family friends, colleagues)

- Formal (issues with psychological counselling, access to supports, organisational pressure)

- Personal (avoidant, anxious etc.)

- Why have these things been unhelpful?

9. Has your trust done anything that makes dealing with exposure to potentially morally injurious events easier?

- Has your trust done anything that makes dealing with exposure to potentially morally injurious events more difficult?

10. Going forward, what measures could be taken by your trust to limit NHS staff experiences of potentially morally injurious events?

- Any measures that you think might be helpful in future crises?

- Any measures needed in general working life

- Systemic changes within the NHS (such as additional training, added regulations, inputting reporting system in the aftermath of PMIES)?

11. Going forward, what do you think could be done differently to help NHS staff cope with the experience of moral injury, if it can’t be avoided?

-Why/how would these things help?

- Are you worried about anything that might be a morally challenging experience for you to deal with in future as we emerge from the pandemic? If so, can you tell me a bit about this?

- What impact might the changes you suggested have on your mental health, responding to future PMIES?

Interview ending

Is there anything we haven’t yet covered that you feel is important to tell me? How did you find the interview? Thank you for talking to me today. I really appreciate your time. The experiences you’ve shared today will be really helpful to understand the effects that moral injury can have on mental health. We hope this information will inform Trusts on how they can best support staff like you and your colleagues. If you would like, we can email you later in the year to update you on our progress and tell you about the key findings from our study. We’ll also send your £25 gift voucher to the email address that we hold for you within 10 days, unless you’d like to provide me with a different email address that you’d prefer me to send it to. If you have any further comments or questions, you can email me at the nhscheck@kcl.ac.uk email address.

## Supplementary File 3

The original themes from the 2021 study

1. Ill equipped and undersupported to respond to crisis

i. Feeling betrayed by the government
ii. Systemic issues within the NHS
iii. Strained working relationships with management
2. Feeling unable to fulfil one’s duty of care towards patients
3. Avoiding moral dissonance
4. Psychological toll of PMIES
i. Anger and guilt
ii. Disillusionment with NHS
iii. Deteriorating mental health
5. Adaptively managing moral distress
i. Resolution of moral distress
